# Suppression of Ongoing Experimental Arthritis by a Chinese Herbal Formula (Huo-Luo-Xiao-Ling Dan) Involves Changes in Antigen-Induced Immunological and Biochemical Mediators of Inflammation

**DOI:** 10.1155/2011/642027

**Published:** 2010-10-19

**Authors:** Ying-Hua Yang, Rajesh Rajaiah, David Y.-W Lee, Zhongze Ma, Hua Yu, Harry H. S. Fong, Lixing Lao, Brian M. Berman, Kamal D. Moudgil

**Affiliations:** ^1^Department of Microbiology and Immunology, University of Maryland School of Medicine, HSF-1, Suite 380, 685 West Baltimore Street, Baltimore, MD 21201, USA; ^2^Mailman Research Center, McLean Hospital, Harvard Medical School, Belmont, MA 02478, USA; ^3^Department of Medicinal Chemistry and Pharmacognosy, University of Illinois at Chicago, Chicago, IL 60612, USA; ^4^Center for Integrative Medicine, University of Maryland School of Medicine, East Hall, 520 W. Lombard St., Baltimore, MD 21201, USA

## Abstract

Rheumatoid arthritis (RA) is one of the major autoimmune diseases of global prevalence. The use of the anti-inflammatory drugs for the treatment of RA is associated with severe adverse reactions and toxicity. This limitation has necessitated the search for novel therapeutic products. We report here a traditional Chinese medicine-based herbal formula, Huo luo xiao ling dan (HLXL), which has potent antiarthritic activity as validated in the rat adjuvant-induced arthritis (AA) model. HLXL (2.3 g/Kg) was fed to Lewis (RT.1^1^) rats daily by gavage beginning at the onset of arthritis and then continued through the observation period. HLXL inhibited the severity of ongoing AA. This suppression of arthritis was associated with significant alterations in the T cell proliferative and cytokine responses as well as the antibody response against the disease-related antigen, mycobacterial heat-shock protein 65 (Bhsp65). There was a reduction in the level of the proinflammatory cytokines IL-17 and IL-1*β* but enhancement of the anti-inflammatory cytokine IL-10 level. In addition, there was inhibition of both the anti-Bhsp65 antibody response and the serum level of nitric oxide. Thus, HLXL is a promising CAM modality for further testing in RA patients.

## 1. Introduction

Rheumatoid arthritis (RA) is one of the major human autoimmune diseases affecting about 1 per cent of the adult population [1, 2]. The disease is characterized by inflammation of the synovial tissue and damage to the cartilage and bone of the joints, leading to severe deformities [1, 3–5]. The precise etiologic agent in RA is not yet defined. The drugs that inhibit inflammatory reactions are a vital component of the therapeutic arsenal against RA [1, 6, 7]. However, the adverse reactions and toxicity associated with the use of these drugs have expeditiously promoted the use of natural plant products or procedures belonging to the diverse traditional systems of medicine by patients with RA [8–14] and other chronic inflammatory disorders [15–23]. This growing trend warrants a continuous search for new natural antiarthritic complementary and alternative medicine (CAM) products with well-defined mechanisms of action. 

Traditional Chinese medicine (TCM) offers a variety of herbal CAM products that have been used in the treatment of patients with chronic inflammatory disorders for several decades [8–19]. However, many of these products have not been validated experimentally for their composition and/or mechanism of action. Huo luo xiao ling dan (HLXL), a TCM-based Chinese herbal formula, has been in use in China for the treatment of patients with a variety of disorders, including arthritis [24, 25]. We have previously reported that HLXL consisting of 11 herbs has anti-inflammatory activity as evident upon testing in an experimental model of paw edema [24, 25]. In this study, based on the rat adjuvant-induced arthritis (AA) model of human RA [26, 27], we report the immunological basis of the therapeutic effect of HLXL against autoimmune arthritis. 

AA can be induced in Lewis (LEW) rats (RT.1^l^) by immunization with heat-killed *M. tuberculosis* H37Ra (Mtb) in oil [26–28]. The mycobacterial heat-shock protein 65 (Bhsp65) is one of the major antigenic targets of the T cell response of arthritic rats [28–31]. Our results show that HLXL suppresses the severity of ongoing inflammatory AA. Furthermore, this antiarthritic activity of HLXL is associated with changes in both the T cell and the antibody responses against Bhsp65. Also altered was the level of serum nitric oxide (NO), a biochemical mediator of inflammation. We suggest that HLXL be considered for further evaluation of its efficacy as a CAM modality for the treatment of RA patients.

## 2. Materials and Methods

### 2.1. Animals

Male, 5- to 6-week-old Lewis (LEW/Hsd) (RT.1^l^) rats purchased from Harlan Sprague-Dawley (HSD) (Indianapolis, IN, USA) were used in this study. This study protocol was approved by the University of Maryland School of Medicine, Baltimore (UMB) and Institutional Animal Care Use Committee (IACUC). The IACUC guidelines follow the Public Health Service (PHS) Policy on Humane Care and Use of Laboratory Animals and the National Research Council Guide for the Care and Use of Laboratory Animals. Furthermore, UMB is fully accredited by the Association for Assessment and Accreditation of Laboratory Animal Care (AAALAC) International.

### 2.2. Antigens

Recombinant mycobacterial heat-shock protein 65 (Bhsp65) was prepared as described elsewhere [32]. Ovalbumin (Ova) was obtained from Sigma-Aldrich (St. Louis, MO), and purified protein derivative (PPD) was purchased from Mycos Research (Fort Collins, CO).

### 2.3. Induction and Evaluation of Adjuvant Arthritis (AA)

AA was induced in LEW rats by s.c. injection at the base of the tail of 1 mg/rat heat-killed *M. tuberculosis* H37Ra (Mtb) (Difco, Detroit, MI) in 200 ***μ***L of mineral oil (Sigma-Aldrich). The severity of arthritis was graded on the basis of erythema and swelling as previously described [32, 33]. The highest score for each paw was 4, and the total maximum score for each rat was 16. The natural course of AA includes 4 phases: incubation (d 0–8), onset (d 9–11), peak (d 17–19), and recovery (d 21–26).

### 2.4. Histopathological Evaluation of Hind Paw Joints of Rats

Hind paws of rats were harvested, skinned, and fixed for 3–7 days with 10% phosphate-buffered formalin (Fisher Scientific, Fair Lawn, NJ). Thereafter, the paws were decalcified in formic acid (Fisher Scientific), embedded in paraffin, and sectioned longitudinally (820 Microtome, Fisher Scientific). Tissue sections (5-*μ*m) were mounted on a glass slide, stained with hematoxylin-eosin (Sigma-Aldrich), and assessed for morphological changes and cellular infiltrates [34].

### 2.5. Composition, Characterization, and Toxicity of the CAM Modality, HLXL

The composition of the herbal formula huo luo xiao ling dan (HLXL), the method for its preparation, and the assessment of toxicity of HLXL have been described previously [24, 25]. The following 11 herbs comprise HLXL: Ruxiang (*Boswellia carterii* Birdw.), Qianghuo (*Notopterygium incisum* Ting ex H.T. Chang), Danggui (*Angelica sinensis* (Oliv.) Diels), Baishao (*Paeonia lactiflora* Pall.), Gancao (*Glycyrrhiza uralensis* Fisch.), Yanhusuo (*Corydalis yanhusuo* W.T. Wang.), Danshen (*Salvia miltiorrhiza* Bge.), Chuanxiong (*Ligusticum chuanxiong* S.H. Qiu.), Qin jiao (*Gentiana macrophylla* Pall.), Guizhi (*Cinnamomum cassia* Presl.), and Duhuo (*Angelica pubescens* Maxim.). The details of the acquisition, authentication, purity, processing, and extraction of the individual herbs, HPLC fingerprinting of HLXL mixture, information on voucher samples and pharmacopoeia, and toxicity are described elsewhere [24, 25]. The toxicity of HLXL was assessed by daily feeding of HLXL to LEW rats for 6 consecutive weeks and then observing these rats regularly for unusual behavioral changes and standard signs and symptoms of toxicity [25]. Blood biochemistry as well as full necropsy and histopathological examination of tissues was also performed to assess toxicity. The control rats were fed with vehicle instead of HLXL. Taking all these parameters together, no adverse reactions or toxicity of HLXL were observed either at 2.3 or 4.6 g/kg [25].

### 2.6. Treatment of Rats with HLXL

The rats were randomly divided into three groups following the onset of arthritis on day 11 after Mtb injection. The experimental group of rats was fed HLXL (2.3 g/kg) daily by gavage beginning on day 12 after Mtb injection and then continued throughout the course of AA. This dose was selected after a pilot study in which 3 doses (g/kg) of HLXL were tested: 1.15, 2.3, and 4.6. The control group of rats received the vehicle (water) by gavage on the corresponding days. A third group of arthritic rats was fed methotrexate (MTX) (Sigma) (0.5 mg/kg), an established antiarthritic compound, as a positive control. All these rats were observed regularly for signs of arthritis.

### 2.7. Lymph Node Cell (LNC) Proliferation Assay

The draining lymph nodes (inguinal, iliac, and popliteal) of rats were harvested on day 7 after the initiation of HLXL feeding. A single-cell suspension of lymph node cells (LNCs) from rats of the respective group (experimental versus control) was prepared separately and then cultured (5 × 10^5^ cells/well of a 96-well plate) in serum-free HL-1 medium (BioWhittaker, Walkersville, MD). The cells were plated in replicates (*n* = 4) and cultured with antigen for 2 days as described in detail elsewhere [33, 35]. Thereafter, ^3^[H]-thymidine (1 *μ*Ci/well) was added to these LNCs and the cells cultured for another 16 h [33, 35], and then the cells were harvested onto a printed glass fiber filter. The incorporation of radioactivity in proliferating cells was measured using a liquid scintillation counter. The results were expressed either as counts per minute (cpm) or as a stimulation index (the ratio of cpm in the presence of antigen and cpm of cells in medium alone). Bhsp65 (6.5 *μ*g/ml) was used as the test antigen, while PPD (30 *μ*g/ml) and Ova (40 *μ*g/ml) served as positive and negative controls, respectively. The indicated optimal concentrations of different antigens used were selected after dose titrations in preliminary experiments.

### 2.8. Isolation of Synovium-Infiltrating Cells (SIC) and the Preparation of Cell Lysate

The ankle region of an arthritic rat was nicked in the metatarsal area. After removal of the skin and superficial tendons, the articular cavity was opened up from the lateral side. Thereafter, the synovial tissue was dissected out into the culture medium placed in a petri dish. This dissected tissue was digested for 1 h at 37°C in Hanks' balanced salt solution (HBSS) containing 3 mg/mL collagenase 1A (Sigma-Aldrich), 3 mg/mL hyaluronidase IV-S 1 mg/mL (Sigma), 0.1 mg/mL DNase II (Sigma), 1% fetal bovine serum (FBS) (Invitrogen), and 5% HEPES (EM Science). The digested tissue was then filtered through a nylon mesh and washed extensively with PBS. The collected cells were lysed in 250 *μ*l lysis buffer (Bio-rad), and the supernate was harvested for testing of cytokine levels.

### 2.9. Testing of the Cytokine Response

**(A) Quantitative Real-Time Polymerase Chain Reaction (qRT-PCR)**

The LNCs were restimulated with antigen for 48 h as in an LNC proliferation assay. The total RNA extracted from these LNCs was reverse-transcribed to cDNA and then tested in qRT-PCR using specific primers for the genes encoding the rat IFN-*γ*, IL-10, and IL-17 employing platinum SYBR Green [36, 37]. The levels of cytokine mRNA were normalized to that of the hypoxanthine-guanine phosphoribosyltransferase gene (*hprt*), and the results were presented as the relative gene expression levels.

**(B) Enzyme-Linked Immunosorbent Assay (ELISA)**

The supernates of lysed synovium-infiltrating cells (SIC) were assayed by ELISA for TNF-*α* and IL-1*β* in the cytokine core facility of UMB. The cytokine levels were normalized to the total protein content which was measured by Bio-Rad Protein Assay (Bio-Rad Laboratories, Hercules, CA).

### 2.10. Measurement of the Level and Isotype of Anti-Bhsp65 Antibodies

The sera of the test and control group of rats (*n* = 3 each) were added at a dilution of 1 : 100 each to antigen-coated wells (100 ng/well) of a high-binding ELISA plate (Greiner Bio-One) [35, 38]. The plate was incubated for 1 h at room temperature, and then the wells were washed thoroughly. The plate-bound total Ig or the IgG1/IgG2a isotype was detected by using the appropriate horseradish peroxidase-conjugated goat antirat antibodies (Zymed). The color intensity was read as optical density (O.D.) at 450 nm using a Vmax ELISA autoreader (Molecular Devices) [35, 38].

### 2.11. Determination of the Serum Nitric Oxide (NO) Levels

The levels of NO in sera of the experimental and control rats were assessed by measuring the nitrite (NO_2_^−^) and nitrate (NO_3_^−^) content using a colorimetric assay kit (Biovision research products, Mountain View, CA) [34]. The results were expressed as mM.

### 2.12. Statistical Analysis

The data were expressed as mean ± SEM. Student's *t*-test and two-way ANOVA were used to assess the significance of differences using GraphPad Prism version 4.0. A *P*-value less than  .05 was considered significant.

## 3. Results

### 3.1. Attenuation of the Severity of Established Adjuvant Arthritis in HLXL-Fed LEW Rats

**Figure 1:**
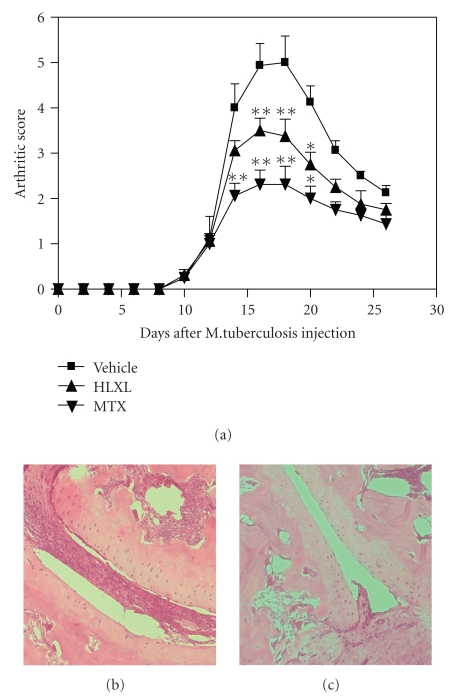
HLXL feeding reduced the progression and severity of ongoing AA in the LEW rat. Arthritic LEW rats were fed by gavage daily with HLXL (2.3 g/kg body weight, experimental group), water (negative control group), or MTX (0.5 mg/kg, positive control group), from day 12 to day 23 after Mtb immunization. Rats were observed regularly throughout the course of AA. Arthritic scores (mean ± SEM) (a) of a representative experiment (*n* = 4 per group) are shown. The days of significant difference in the arthritic scores of HLXL/MTX group versus the control are marked (*, *P* < .05; **, *P* < .01). Also shown are representative hind paw histopathology sections of water-fed control (b) and HLXL-fed (c) rats on day 18.

The signs of inflammatory arthritis appeared about 10 days after Mtb injection of the LEW rats. Accordingly, HLXL, MTX, or water was administered daily to rats from day 12 to day 23 of Mtb injection. The results (Figure 1(a)) show that both the HLXL-fed and the MTX-fed LEW rats had significantly reduced arthritic scores compared to that of the water-fed control rats. The reduction in the severity of arthritis was significant between day 16 and day 20 (*P* < .01 at day 16 and day 18, *P* < .05 at day 20) for the HLXL group (test group), and between day 14 and day 20 (*P* < .01 at day 14, day 16 and 18, *P* < .05 at day 20) for the MTX group (positive control group). However, the suppressive effect of HLXL on arthritis was relatively less marked than that of MTX (*P* < .01 at day 16, *P* < .05 at day 16). Histopathological analyses of the hind paws showed that on day 18, the extent of the inflammatory cell infiltration as well as bone and cartilage destruction was much less in HLXL-fed rats (Figure 1(c)) than that in the control rats (Figure 1(b)).

### 3.2. HLXL Diminished the T Cell Proliferative Response and Deviated the Cytokine Response to Bhsp65 in Arthritic LEW Rats

**Figure 2:**
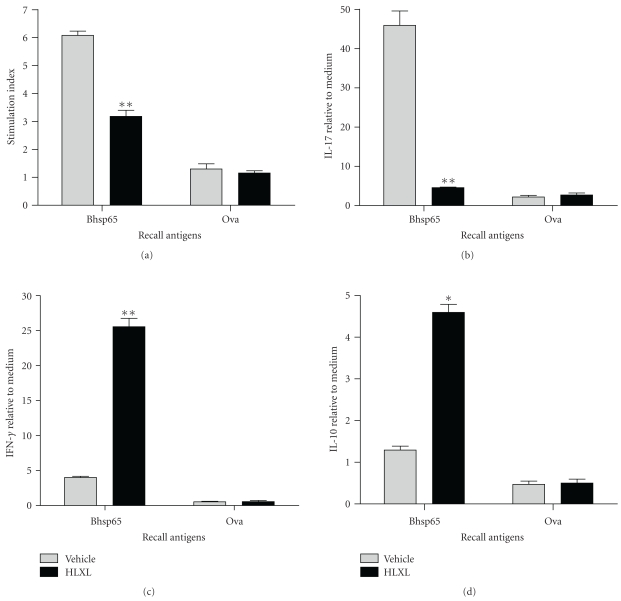
Alteration of the T cell proliferative and cytokine responses of HLXL-treated arthritic rats. LNCs of arthritic rats harvested on d 7 after initiation of the daily feeding of HLXL or water were tested for their T cell proliferative (a) and cytokine response (b)–(d) to antigenic restimulation with Bhsp65 in vitro. Ova served as the control antigen. The results of the proliferation assay were expressed as mean stimulation index (SI) ± SEM. The cpm value for PPD was 9,240 (the control group) and 5,577 (HLXL group). For cytokines, the level of mRNA for IL-17 (b), IFN-*γ* (c), and IL-10 (d) was measured by qRT-PCR, normalized to HPRT expression, and compared with that of cells in medium. The results of a representative experiment (*n* = 3 per group) are shown. The data are expressed as “fold relative to cells in the medium”. (*, *P* < .05; **, *P* < .01).

 We next investigated the immunological changes associated with the decreased severity of AA following HLXL treatment. We tested the Bhsp65-specific proliferative and cytokine responses of the draining LNCs of arthritic LEW rats. The HLXL-fed rats showed a significantly (*P* < .001) reduced T cell proliferative response to Bhsp65 compared to that of the control rats (Figure 2(a)). Furthermore, HLXL feeding suppressed the proinflammatory IL-17 (Figure 2(b)) (*P* < .01), but increased both the anti-inflammatory IL-10 (Figure 2(d)) and the proinflammatory IFN-*γ* (Figure 2(c)) production.

### 3.3. Suppression of the Antibody Response in Rats Treated with HLXL

**Figure 3:**
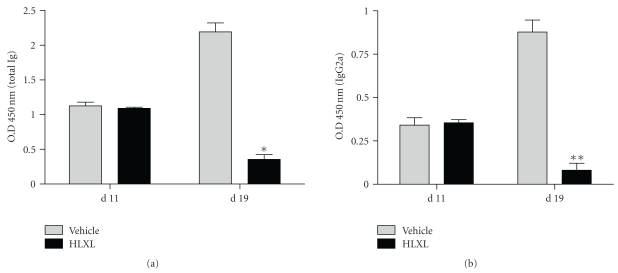
HLXL inhibited the antibody response to Bhsp65 of LEW rats with AA. Blood samples were collected from experimental (HLXL-fed) and control (water-fed) groups of rats (*n* = 3 per group) at the indicated time points, and then the sera were tested at a dilution of 1 : 100 each by ELISA for total Ig (a) and IgG2a (b). The results were expressed as optical density (O.D.) at 450 nm (mean ± SEM). (*P < .05).

We also measured the serum levels of total Ig as well as isotype-specific IgG2a and IgG1 anti-Bhsp65 antibodies at different time points after the onset of arthritis. The levels of both the total immunoglobulins (Ig) and the IgG2a antibodies against Bhsp65 in HLXL-fedats versus the levels of the respective antibody type in the water-fed controls were comparable on d 11, but significantly lower on d 19 (*P* < .01 for total Ig, *P* < .05 for IgG2a) (Figures 3(a) and 3(b)). The level of IgG1 was barely above the background (data not shown).

### 3.4. HLXL-Mediated Reduction in the Level of IL-1*β* in the Target Organ-Infiltrating Cells in Arthritic Rats

**Figure 4:**
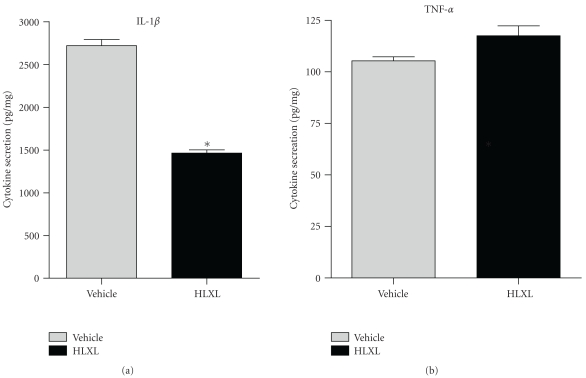
Treatment with HLXL suppressed IL-1*β* production by the synovium-infiltrating cells of arthritic rats. The levels of IL-1*β* (a) and TNF-*α* (b) in SIC harvested from the water-fed and HLXL-treated rats (*n* = 3 per group) on d 19 after Mtb injection were determined by ELISA. The results were expressed as pg/mg. (*P < .05).

To study the HLXL-induced in vivo modulation of the inflammatory response, the SICs were collected from the joints of HLXL-fed rats and water-fed rats on d 19 after Mtb injection and then tested for cytokine secretion. HLXL treatment significantly inhibited IL-1*β* levels by 42% (*P* < .001), but without any change in the level of TNF-*α* compared to that of the water-fed group (Figure 4).

### 3.5. Rats Treated with HLXL Revealed Reduced Levels of Serum Nitric Oxide (NO)

**Figure 5:**
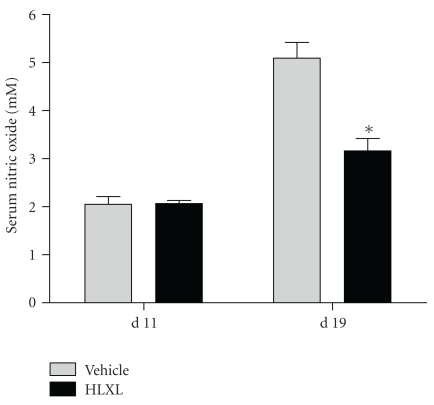
The influence of HLXL on the level of serum NO in arthritic LEW rats. Sera were obtained from HLXL-fed and water-fed rats (*n* = 3 per group) on the indicated days as described under Materials and Methods. The level of NO in these samples was determined by a colorimetric assay. The results were presented as mM (mean ± SEM). (*P < .05).

Sera of HLXL-fed rats showed reduced level of NO at d 19 (*P* < .05) (Figure 5), and this reduction in NO level showed an association with a reduction in the severity of arthritis. 

## 4. Discussion

The results of this study, based on the rat AA model, have revealed that HLXL, a traditional Chinese medicine-based herbal formula, can suppress the ongoing disease. The antiarthritic activity of this CAM modality was associated with significant changes in the T cell proliferative and cytokine profile of the draining lymph node cells (LNCs), the cytokine secretion by the synovial tissue-derived cells, the antibody response to Bhsp65, and the level of serum nitric oxide (NO). 

Arthritic LEW rats raise T cell response to Bhsp65 during the course of AA [28–32]. The Mtb-primed pathogenic T cells include subsets of T cells reactive against Bhsp65 [27, 39, 40]. It is conceivable that an antiarthritic compound might limit the development of arthritis in part by reducing the activation and expansion of Bhsp65-reactive T cells. In fact, HLXL-fed LEW rats showed reduced T cell proliferative response to Bhsp65 compared to the control water-fed rats. In addition, HLXL feeding induced a significant alteration in the cytokine response of Bhsp65-reactive T cells that facilitated the inhibitory effect of the herbal mixture on inflammatory arthritis. The majority of the literature on AA is based on Th1 type of arthritogenic response [27, 39]. Accordingly, a Th2 type of response would be counter-regulatory for the Th1 type of response [41–43]. In addition, in the last decade, the role of IL-17 in the pathogenesis of arthritis is increasingly being realized [44–46] through studies in the animal models of arthritis, including AA [37, 47–49] as well as patients with RA [50–52]. In view of the above cytokine-mediated events in the pathogenesis of autoimmune arthritis, it is anticipated that a potential antiarthritic compound might skew the cytokine milieu towards a predominantly anti-inflammatory type [41–43, 46]. This can be achieved by enhancing the Th2 response and/or reducing the pathogenic Th1/Th17 response. Our results showed that feeding of HLXL to arthritic LEW rats indeed enhanced the Th2 (IL-10) response and concomitantly reduced the Th17 (IL-17) response. However, there also was an unexpected increase in the Th1 (IFN-*γ*) response. This cytokine balance was unanticipated in the context of the Th1-Th2 paradigm of cytokine-based regulation [41–43, 46], which would have predicted a reduced IFN-*γ* coupled with an enhanced IL-10 leading to deviation of the cytokine response to a predominantly Th2 type. Nevertheless, the observed increase in IFN-*γ* matched with the concurrent reduction in IL-17. It has been reported by other investigators that IFN-*γ* can downregulate the IL-17 response [45, 49]. Similarly, a study from our laboratory also revealed that tolerization with Bhsp65 reduced IL-17 production but enhanced IFN-*γ* response [37], supporting the idea of IFN-*γ*-mediated regulation of IL-17. We suggest that the HLXL-induced increase in IFN-*γ* inhibits the IL-17 response, which in turn cooperates with the enhanced IL-10 response to downmodulate the severity of inflammatory arthritis in LEW rats. At present, we do not know the precise mechanism by which HLXL suppresses the IL-17 response. Nevertheless, the results of our current study combined with the recently reported success of a derivative of the plant alkaloid febrifugine in inhibiting the differentiation of Th17 cells and suppressing autoimmunity [53] highlight the significance of defining the mechanisms of action of CAM products for fully exploiting their therapeutic potential. 

The above-mentioned HLXL-induced changes involve the cytokines produced primarily by the T cells. We also examined the effect of HLXL on two of the major arthritis-related cytokines (IL-1*β* and TNF-*α*) produced by the synovial tissue from the joints of arthritic mice and rats [54–57]. Macrophage is one of the main cell types producing these two cytokines. Our results showed that HLXL feeding significantly reduced the levels of IL-1*β* but without much effect on TNF-*α*. Although both of these cytokines have been shown to play a major role in the pathogenesis of RA [3, 57, 58], HLXL feeding induced a differential effect on the two cytokines in AA. IL-1*β* induces a cascade of inflammatory reactions that eventually lead to tissue damage. The blocking of IL-1*β* by immunochemicals (e.g., anticytokine antibody) is being explored as a therapeutic approach for RA [3, 57, 58]. In this regard, the results showing HLXL-induced inhibition of the IL-1*β* production are of significance. 

Arthritic LEW rats develop not only T cell response to Bhsp65 but also antibodies to the same antigen [31, 38]. Antibodies play an important role in the development of arthritis in CIA and K/BXN model of arthritis [59–63], but the role of antibodies in the initiation or propagation of AA is not yet clear. Our results show that HLXL-fed rats had a significant reduction both in the titers of anti-Bhsp65 antibodies and the severity of arthritis on d 19 after disease induction. These results using HLXL in a therapeutic regimen in AA are supported by the results of two of our previous studies based on a preventive regimen for AA, one using green tea [64] and the other using HLXL [65]. We suggest that HLXL has an inhibitory effect on the production of a potentially disease-promoting subset of antibodies, which might mediate their pathogenic effect, for example, via activation of the complement pathway [62, 63]. However, at present, it is not clear whether a reduced antibody response of HLXL-fed rats contributes directly or indirectly to the reduced arthritic severity, or it simply reflects an epiphenomenon whereby HLXL feeding reduces the production of antibodies but without any effect on disease severity. 

HLXL feeding to LEW rats reduced the levels of serum NO which is one of the biochemical mediators of inflammation [55, 66–68]. NO has been shown to have a pathogenic role in the development of arthritis in animal models as well as RA patients [55, 66–68]. In our study, the timing of maximal inhibition of NO levels directly correlated with that of the significant reduction in the severity of AA. However, at present, it is not clear whether NO is the cause or the effect of reduced arthritis severity in HLXL-fed rats. Nevertheless, the results suggest that NO might serve as one of the biomarkers for monitoring the anti-inflammatory effects of HLXL in vivo in the AA model.

We observed suppression of arthritis in rats treated with HLXL beginning on d 12 after disease induction and then continuing daily HLXL feeding throughout the rest of the course of arthritis. Typically, in control rats treated with vehicle alone, the severity of arthritis peaked around d 16 followed by a gradual, spontaneous decline in disease severity after another 3-4 days. Considering the component of spontaneous recovery of clinical arthritis in the AA model, additional investigations would be needed to determine the relative efficacy of HLXL in preventing the development of peak arthritic symptoms owing to an early (starting HLXL feeding on d 12) intervention versus that in suppressing arthritis at the peak phase of the disease following a late intervention (starting on d 16). Similarly, the comparative antiarthritic activity of the herbal mixture versus that of its individual component herbs remains to be determined. HLXL consists of 11 component herbs, some of which have been used individually or in different combinations in TCM for the treatment of arthritis and other inflammatory conditions [24, 25]. One notable component among these is represented by *Boswellia carterii* or a related species and its bioactive chemical constituents, boswellic acids, which we have previously shown to possess anti-inflammatory and antiarthritic activity [69] and to induce cytokine deviation to the Th2 type [70]. It is likely that there are additional chemical constituents besides the boswellic acids within HLXL that not only contribute to its anti-inflammatory and antiarthritic activity, but also help in limiting the toxicity of the herbal mixture. In TCM, the relative proportion of different components of a herbal mixture is aimed at deriving a formula that has optimal efficacy but minimal side effects. Moreover, there might be unique combination effects of the component herbs in a herbal mixture [71, 72]. 

**Figure 6:**
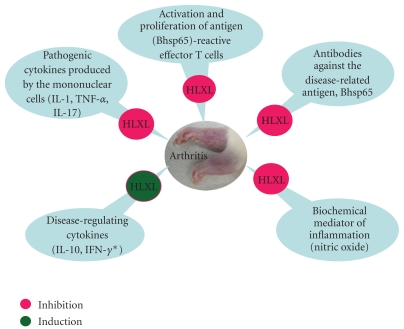
A schematic representation of the diverse mechanisms involved in HLXL-induced immunomodulation of adjuvant arthritis. HLXL suppressed arthritis via the induction of disease-regulating cytokines coupled with the inhibition of pathogenic events that facilitated the initiation and propagation of arthritis. (*IFN-*γ* has a dual role in autoimmunity.)

In summary, HLXL, a traditional Chinese medicine-based CAM modality, has therapeutic antiarthritic activity as tested in the rat AA model. The HLXL-induced inhibition of ongoing AA involved significant changes in the cellular as well as humoral immune responses to the disease-related antigen, Bhsp65 (Figure 6). Notably, HLXL feeding caused suppression of the proinflammatory cytokines IL-17 and IL-1*β* along with upregulation of the anti-inflammatory cytokine IL-10. Also, there was attenuation of the anti-Bhsp65 antibody response and reduction in the level of NO, a known mediator of inflammation. On the basis of our results in the AA model, we suggest that HLXL is a promising therapeutic agent that should be tested further in preclinical trials in RA patients.
